# Retention of nickel, cobalt and chromium in skin at conditions mimicking intense hand hygiene practices using water, soap, and hand-disinfectant in vitro

**DOI:** 10.1186/s12995-024-00442-5

**Published:** 2024-11-06

**Authors:** Libe Vilela, Linda Schenk, Anneli Julander, Klara Midander

**Affiliations:** 1 Institute of Environmental Medicine (IMM), Karolinska Institutet, Stockholm, Sweden; 2 IVL Swedish Environmental Research Institute, Stockholm, Sweden

**Keywords:** Skin, Retention, Penetration, Metals, Hygiene practices, Sodium lauryl sulphate

## Abstract

**Background:**

During the COVID-19 pandemic, increased hand hygiene practices using water, soap and hand disinfectants, became prevalent, particularly among frontline workers. This study investigates the impact of these practices on the skin’s ability to retain the allergenic metals nickel, cobalt, and chromium. The study constitutes three parts: (I) creating an impaired skin barrier, (II) exposing treated and untreated skin to nickel alone, and (III) in co-exposure with cobalt and chromium.

**Methods:**

Using full-thickness skin from stillborn piglets, in vitro experiments were conducted to assess retention of metals in skin at conditions mimicking intense hand hygiene practices. Treatment of skin with varying concentrations of sodium lauryl sulphate (SLS), to impair its barrier integrity was assessed. This was followed by exposure of treated and untreated skin to the metals, that were dissolved in Milli-Q water, 0.5% SLS, and ethanol respectively.

**Results:**

Results showed that pre-treatment with 5% SLS impaired the skin barrier with regards to the measure of trans epidermal water loss (TEWL). Metal amounts retained in the skin were generally higher in treated than untreated skin. The highest amounts of metal retained in skin were observed for exposure to nickel in ethanol. Co-exposure to nickel, cobalt, and chromium in 0.5% SLS resulted in the highest amounts of total metal retention.

**Conclusions:**

The in vitro findings highlight the increased risk of metal retention in skin due to an impaired barrier. The SLS concentration used in the current study corresponds to those used in many hand hygiene products. Hence, occupational settings with frequent exposure to water, soap and disinfectants need to consider protective measures not only for the irritant exposures themselves but also simultaneous exposure to allergenic metals.

**Supplementary Information:**

The online version contains supplementary material available at 10.1186/s12995-024-00442-5.

## Introduction

During the Coronavirus pandemic 2019 (COVID-19), hygiene practices changed and more frequent exposure to water and hand disinfectants was observed [1]. Several studies related to increased hygiene measures during this period, have focused on healthcare workers and how such COVID-19 related measures increased skin- health issues [2–5]. Correspondingly, an increased self-reported exposure to water, soap and usage of hand disinfectant were demonstrated in frontline workers and IT personnel, i.e., occupations outside of the hospital setting that required presence at the workplace and one that could largely work from home [6]. Additionally, frontline workers also reported higher frequency of hand eczema than IT personnel [6], which is in line with the associations between increased hand washing and hand eczema observed for healthcare workers [2–5].

The most common cause of occupational skin disease has previously been reported to be occupational contact dermatitis [7, 8], which depending on aetiology, can be divided into irritant contact dermatitis and allergic contact dermatitis. While water, detergents, and cleansers are among the most important irritants, also having the ability to impair the skin barrier [9–11], the allergenic metals nickel, cobalt and chromium are common causes of occupational contact dermatitis [12]. Simultaneous or consecutive exposure to both irritant/barrier damaging chemicals and allergens is common, not least in wet work occupations [13], including health care workers [14]. In addition, such combined exposures will affect the possibility and degree of penetration and retention of allergens into the skin, although to an unknown extent.

The hypothesis of this study was that increased hand hygiene practices, amidst the COVID-19 pandemic, could lead to an impaired skin barrier, which in turn, might affect the skin barrier’s ability to retain allergenic metals. To test this hypothesis, in vitro experiments were performed in accordance with the OECD Test Guideline for skin absorption [15] to study retention of metals, in different solvents. The aim was to elucidate to what degree skin penetration occurred at exposure, under conditions mimicking intensive hand cleaning with water, soap, hand sanitizer.

## Methods

**Fig. 1:**
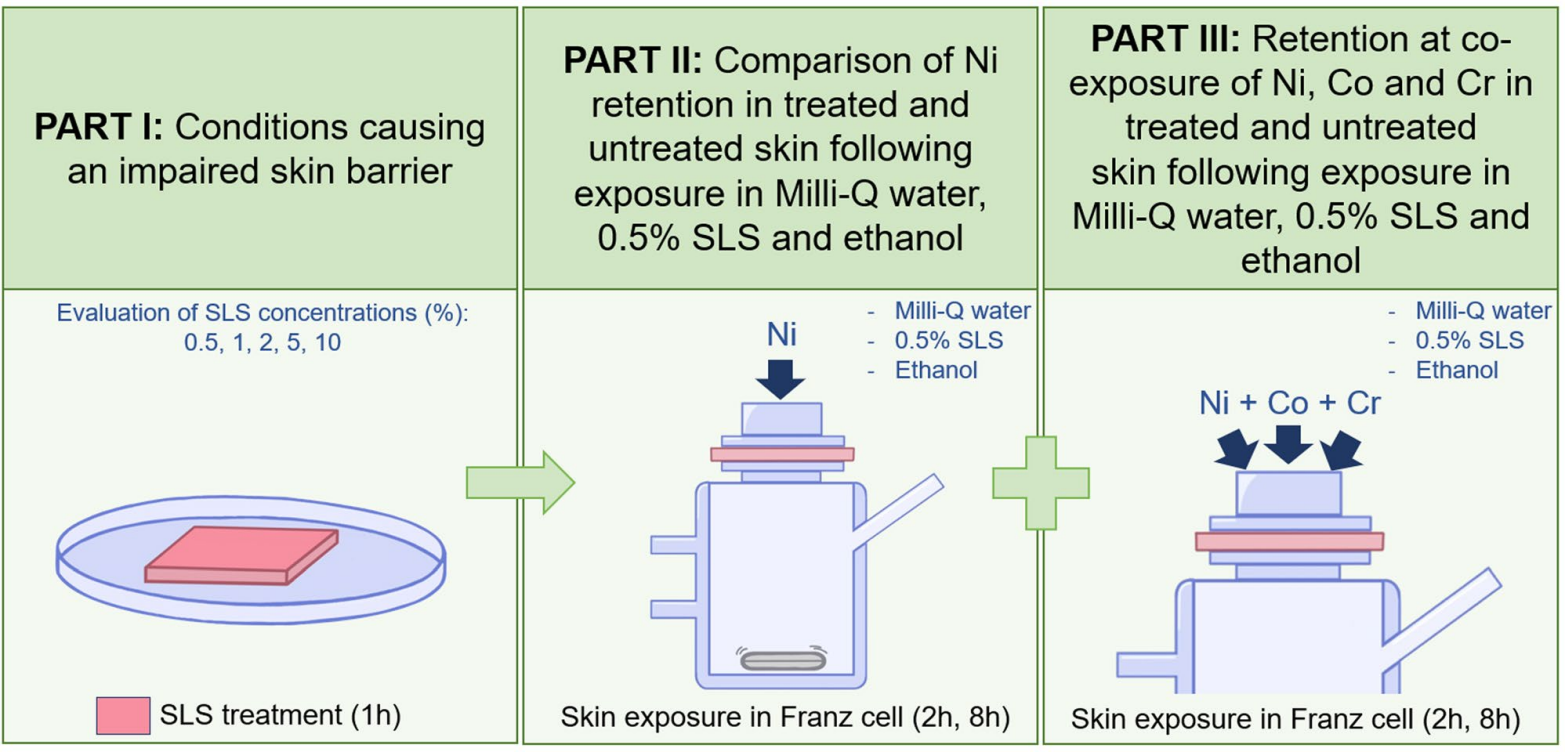
Schematic illustration of the three experimental parts (I, II, III) of the study

In in vitro-experiments to study retention of allergenic metals in skin, conditions mimicking intense hand hygiene practices using water, soap and hand-disinfectant, were obtained by simultaneous exposure to nickel alone or in combination with cobalt and chromium, and the exposure solvents Milli-Q water, 0.5% sodium lauryl sulphate (SLS) and ethanol, respectively. In addition, skin with impaired barrier properties, to further resemble damage from intensive hand hygiene, was created via pre-treatment with SLS. In practical terms, the study was divided into three different experimental parts; *I* - in which conditions causing an impaired skin barrier were tested and evaluated, *II* - in which treated and untreated skin were exposed to nickel in Milli-Q water, 0.5% SLS and ethanol, and *III* - in which treated and untreated skin were co-exposed to nickel, cobalt and chromium in Milli-Q water, 0.5% SLS and ethanol, Fig. 1. (Flowchart of the chronological order of the study can be found in Supplementary Material Figure S1). All material used in experiments were acid washed (soaked for 24 h in 10% HNO_3_, rinsed three times with ultrapure water and dried in ambient laboratory air) or cleaned with ethanol, to avoid any possible metal contamination.

### Skin for experiments

Full-thickness skin of stillborn piglets from commercial breeders was used in the present study. As the animals were not bred for research purposes, the use is exempt from the Swedish Agency for Agriculture’s requirements for ethical vetting of research involving animals. Although the OCED TG 428 does not specify the use of pig skin, the GD 156 [16] state the fact that pig skin is considered an appropriate alternative to human skin, which is also in line with the results from a review of in vitro penetration studies by Barbero et Frasch [17]. Pig skin is routinely used in skin permeation assays as it has been shown to have similar permeability characteristic to human skin [17–20]. Stillborn piglet skin has also been reported to have comparable permeability to human skin for organic compounds [21, 22]. No data is available regarding its metal permeability, but it has been used in other studies of metal retention, for nickel, cobalt, chromium and lead [23, 24].

At arrival to the laboratory, stillborn piglets were rinsed with lukewarm water, after which skin integrity was checked by measuring the transepidermal water loss (TEWL, Dermalab, Cortex Technology, Hadsund, Denmark).

To simulate experimental exposure conditions affected by intense hand hygiene practices, the skin of stillborn piglets was washed with water and soap for 5 min (DAX Mildtvål Oparfymerad, KiiltoClean, Hyllie Stationstorg 2, Malmö, Sweden) or repeatedly treated 25 times with hand disinfectant (DES 75 vol%, LIV by Clemondo, Helsingborg, Sweden) in situ. Based on the results from TEWL measurements following each step in the procedure, the approach was concluded to not efficiently impair the skin barrier and hence, were not used for the experiments (for more information on TEWL values and the procedure see Supplementary Material TableS1).

Full thickness skin (mean thickness 0.86 ± 0.22 mm) was collected from the back and flank of the stillborn piglets (< 24 h post-mortem) and the effect of excision on the skin was checked measuring TEWL at four different locations in each skin piece. Skin thickness was measured with a digital micrometre (model number 293-666-20 Mitutoyo, Kawasaki, Japan). An average TEWL ≥ 11 g⋅m^− 2^⋅h^− 1^ was used as a cut-off for inclusion [25, 26]. However, no skin pieces had to be discarded. The average TEWL for the skin was 7.25 ± 1.22 g⋅m^− 2^⋅h^− 1^. Next, the skin was wrapped in polyethene film and aluminium foil and stored at − 20ºC until later use within 3 months.

On the day of experiments, using a sterile scalpel (Kiato, Sylak AB, Askim, Sweden) 3 × 3 cm skin pieces were cut from each frozen skin and placed in a petri dish to thaw for 30 min at room temperature. Thereafter, the barrier integrity of each skin piece was controlled. The measured TEWL of all skin samples were < 11 g⋅m^− 2^⋅h^− 1^.

### Treatment of skin with SLS (I)

Pre-treatment of skin to alter the barrier integrity can be performed by physical means [27], but for the purposes of this study, a pre-treatment with aqueous SLS was elaborated based on the OECD TG 439 for in vitro skin irritation [28].

After the thawing of skin, 500 µl PBS (PBS, pH = 7.4, Gibco Life Technologies, Thermo Fisher Scientific, Waltham, MA, USA) was put in the petri dish underneath the 3 × 3 cm skin piece to prevent dehydration. The skin surface was exposed to 200 µl of SLS-solutions (diluted from 20% SLS in H_2_O, Sigma-Aldrich, Schnelldorf, Germany) at different concentrations; 0.5, 1, 2, 5, and 10% in Milli-Q water (18.2 MΩ ⋅ cm^− 1^, Merck Millipore, Darmstadt, Germany) for 1 h, covered by the petri dish lid [29]. The concentrations were chosen based on available literature where 0.5-2% SLS concentrations has been used to irritate human skin [11, 30, 31]. OECD test guidelines for in vitro skin irritation using reconstructed human epidermis suggest the use of 5% aqueous SLS as positive control [28]. Due to the different skin models used in the available literature, and to ensure that we select the SLS concentration with the highest effect on the skin barrier, we decided to test also 10%, a concentration above those reported in the previous literature. The SLS was removed by rinsing with 4 ml (2 ml per side) of deionized water (dH_2_O, 16.8 MΩ ⋅ cm^− 1^). In total, four replicate samples were produced for each concentration tested with skin originating from four different piglets. The experiments for the two highest concentrations 5 and 10% respectively, were repeated and the results are thus based on 8 replicate samples. The TEWL values for each skin sample was recorded 20 min after removing the treatment.

### Franz diffusion cell experiments (II, III)

A series of experiments were conducted to evaluate the ability of the skin barrier to retain metals given conditions without and with SLS pre-treatment, to alter the skin barrier, and the simultaneous exposure to Milli-Q water, 0.5% SLS and ethanol, to mimic intensive hand hygiene practices using water, soap and hand sanitizer, respectively. The OECD TG 428 for skin absorption [15] and GD 156 [16] constituted the starting point for experiments with a focus on the study of the skin barrier as boundary for exposure.

Six jacketed Franz cells (orifice diameter 11.28 mm, corresponding to an exposure area of 0.95 cm^2^, receptor volume 3 ml, Permegear, Bethlehem, PA, USA) were mounted on an adapted magnetic stirrer plate (HP 6 Variomag, H + P Labortechnik, Munick, Germany) and by means of circulating water from a thermostat water bath (AT 110, Heto, Alleod, Denmark) the diffusion cells were tempered at 32ºC. PBS was used as receptor fluid and was kept stirred using Teflon coated magnetic stirring bars. Skin pieces were mounted onto the Franz cells 15 min before the start of metal exposures.

This study comprises twenty-four different exposure scenarios each tested on both treated and untreated skin (Table 1), with a dose range of relevance for occupational settings and exposure time that mimic real-life work periods (short exposure and full day work shift) [32–34]. In the experimental part II, skin was exposed to nickel (1.36 µmol corresponding to a dose of 80 µg Ni/cm^2^) dissolved in Milli-Q water, 0.5% SLS and ethanol (≥ 96%, v/v, TechniSolv^®^, France) for 2 and 8 h. In part III, skin was similarly co-exposed to equimolar amounts of nickel, cobalt and chromium (4.09 µmol corresponding to a dose of 80 µg Ni + 80 µg Co + 71 µg Cr/cm^2^) in the three exposure solvents. The donor solutions were prepared using two metal reference materials: a standard nickel stock solution (10 000 µg Ni/ml in 2.5% HNO_3_, Spectrascan, Teknolab, Ski, Norway) and a special, equimolar high concentration reference material of nickel + cobalt + chromium (Ni + Co + Cr 200 mmol/l in 10% HNO_3_, Spectrascan, Teknolab, Ski, Norway).

Once the skin exposures to metal were initiated, the donor compartment and sampling port were occluded with parafilm (PARAFILM^®^, American National Can™). Blank (Milli-Q) exposures were carried out in parallel to enable control for any metal baseline quantities found in the skin (Supplementary Material Figure S2).

**Table 1:** Exposure experiments included in the current study. Piglet skin was classified as treated when it was exposed to 5% SLS for 1 h, and it was classified as untreated when it was left intact. The metal exposures were either nickel alone or in co-exposure with cobalt and chromium. Metals were dissolved in three types of exposure solvents (Milli-Q water, 0.5% SLS or ethanol), and per type of solvent the skin from six different piglet individuals was used (*n* = 6). Exposure time was 2–8 h. experiments shown in the table were performed for both nickel single exposure and co-exposure to nickel, cobalt and chromium. Including blank samples, a total of 48 different experiments were run, resulting in 288 Franz diffusion cells in total, and the skin of 36 different piglet individuals (*N* = 36) were used

		Metal(s) dissolved in		
		Milli-Q (*n* = 6)	0.5% SLS (*n* = 6)	Ethanol (*n* = 6)
Piglet skin	Treated	2 h, 8 h	2 h, 8 h	2 h, 8 h
	Untreated	2 h, 8 h	2 h, 8 h	2 h, 8 h

### Metal quantification

Post exposure, the skin surface was rinsed with 2 ml dH_2_O per side (4 ml in total). Biopsy punches (Kai medical, 8 mm diameter) were taken from the exposed area and placed in polypropylene-plastic tubes (12 ml, Sarstedt, Nümbrecht, Germany) with 1 ml of 67% HNO_3_ for 48 h (until fully digested). Prior to metal analysis, 50 µl of digested skin was diluted with 4.95 ml of dH_2_0 and spiked with 20 µl of indium (1.255 µg In/ml, diluted from stock solution of 999 ± 5 µg In/ml in 2% HNO_3_, Spectrascan, Teknolab, Ski, Norway).

Quantitative analyses of Ni, Co and Cr were performed using Inductively Coupled Plasma-Mass Spectrometry (ICP-MS iCAP Q Thermo Fisher Scientific, Qtegra version 2.10). Concentrations of ^58^Ni, ^60^Ni, ^59^Co, and ^52^Cr, were analysed in kinetic energy discrimination (KED) measurement mode using helium gas to reduce any polyatomic interference and argon as nebulizer gas, cool gas, and auxiliary gas.

Matrix-matched standards for calibration with the concentrations of 0, 0.1, 1, 5, 10, 50, 100 and 500 µg/l Ni, Co, Cr and Pb in 2% HNO_3_ (67–69% HNO3, VWR, Normatom, Leuven, Belgium) were diluted from single metal reference materials (Ni: 1001 ± 4 µg/ml in 2% HNO_3_ (v/v); Co: 1000 ± 3 µg/ml in 3% HNO_3_ (v/v); Cr: 1002 ± 4 µg/ml in 2% HNO_3_ (v/v); Pb: 998 ± 4 µg/ml in 0.5% HNO_3_ (v/v), Spectrascan, Teknolab, Ski, Norway).

To ensure statistical certainty, each sample was analysed three to five times. The limit of detection (LOD) (based on 7 concentration points of the STD curve in the ICP-MS) was set at 0.079 µg/l for ^58^Ni, 0.082 µg/l ^60^Ni, 0.004 µg/l ^59^Co, and 0.19 µg/l ^52^Cr. All exposed samples analysed were above LOD. Nickel quantities found in samples was calculated as an average of ^58^Ni and ^60^Ni.

### Statistical analysis

Any statistical relationship between the amount of metal retained in skin at exposures to nickel alone or in combination with cobalt and chromium in three exposure solvents for treated and untreated skin at two different time-points were evaluated using the Mann-Whitney U-test (GraphPad Prism version 9.5.0).

To determine which variable (TEWL, skin thickness, +/- SLS treatment, single nickel or Ni + Co + Cr co-exposure in Milli-Q water, 0.5% SLS or ethanol, and exposure time) affect metal retention in skin, linear regression with log-transformed amount of retained metal was performed using R (Version 4.4.1 (2024-06-14 ucrt)).

## Results

### Conditions causing an impaired skin barrier (I)

The median of TEWL values recorded after each step in the preparation of skin for experiments, and after treatment with five different aqueous SLS concentrations (0.5%, 1%, 2%, 5% and 10%) respectively, are compiled in Table 2. A ∆TEWL was calculated from the difference between the measured TEWL value after freezing (post thawing) and the TEWL value after SLS treatment. The results show that among the tested concentrations, 5% aqueous SLS alters the skin barrier the most.

**Table 2:** Median TEWL values recorded (g⋅m^− 2^⋅h^− 1^) for each step (a-d) of the skin preparation procedure including SLS treatment at different concentrations. The TEWL was measured four times at different locations on each skin piece immediately after each step of the skin preparation procedure (a-b) and three times 30 min after thawing the skin (c) and 20 min after removing the SLS treatment (d). The range of these repeated measurements, somewhat indicative of intra- and inter individual variations, is represented by min and max values

Piglet identification			PIG 1	PIG 2	PIG 3	PIG 1 & 4	PIG 2 & 4
			Median (min; max)	Median (min; max)	Median (min; max)	Median (min; max)	Median (min; max)
*Preparation of skin for experiment*							
a)		After rinsing with lukewarm water in situ	11.35 (5.8; 15)	8.10 (6.8; 12.2)	4.25 (3.1; 6.1)	4.85 (2.9; 15.0)	5.35 (2.9; 9.0)
b)		Excised skin	4.55 (4.3; 5.3)	9.20 (7.9; 9.8)	4.10 (3.8; 4.4)	5.15 (4.3; 7.4)	7.65 (5.0; 9.8)
*Skin stored in freezer for up to 3 months*							
c)		After thawing	7.15 (6.6; 7.8)	8.75 (6.8; 10.7)	6.95 (6.2; 8.5)	7.40 (5.5; 9.4)	6.90 (6.0; 10.8)
		***SLS concentration***	0.5%*	1%*	2%*	5%**	10%**
d)		After SLS treatment	11.20 (7.8; 14.8)	10.75 (9.4; 13.4)	9.15 (7.7; 10.8)	13.10 (9.3; 31.0)	11.10 (9.4; 26.2)
		**∆TEWL (d-c)**	**4.05**	**2.00**	**2.20**	5.70***	**4.20**

*skin from one piglet for each concentration, four samples, *n* = 4

**skin from two different piglets for each concentration, eight samples, *n* = 8

***SLS 5% was chosen to treat skin samples with for Part II and Part III, since it produced the highest change in ∆TEWL

### Skin exposure to nickel in Milli-Q water, 0.5% SLS and ethanol (II)

**Fig. 2:**
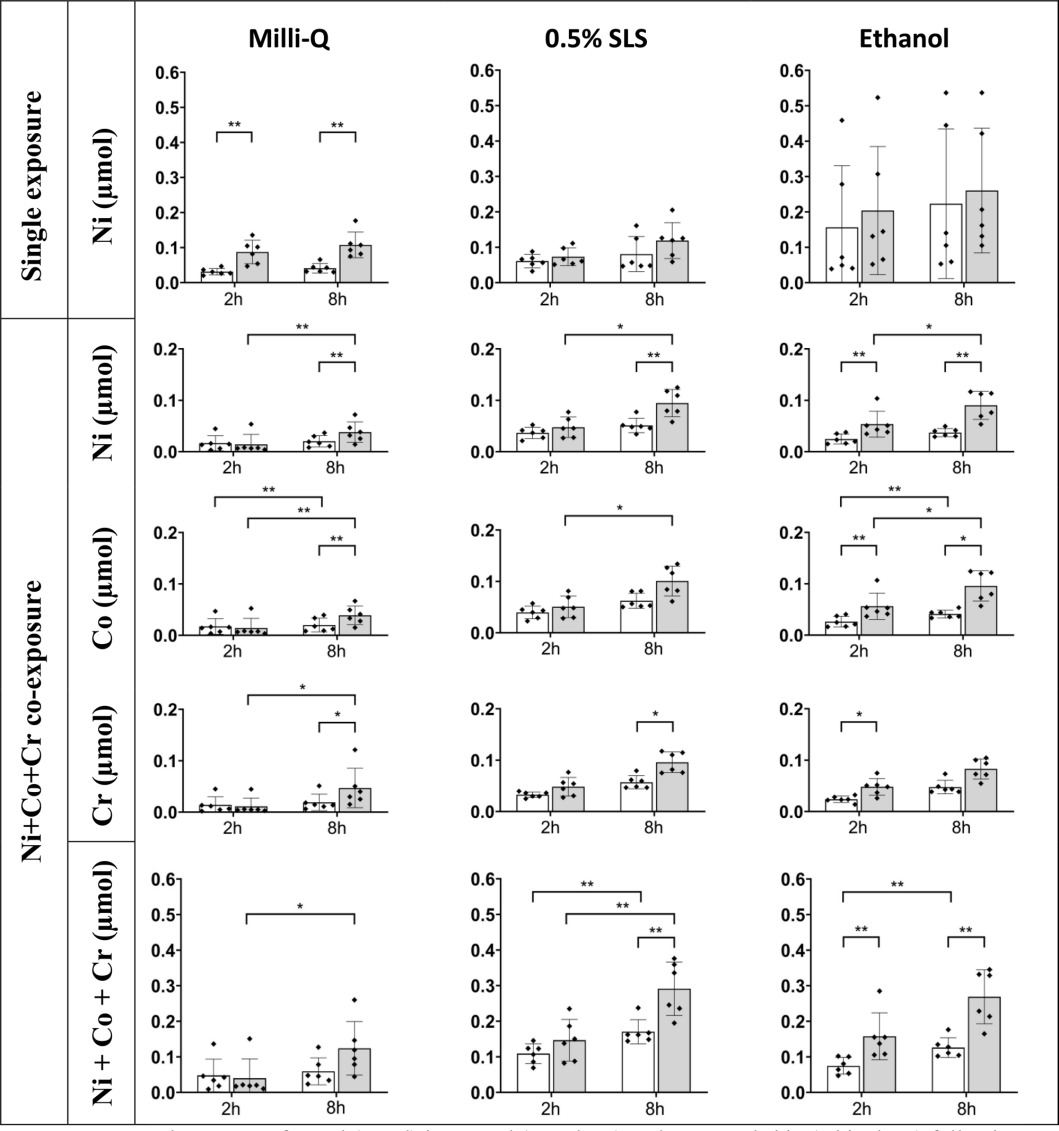
Measured amounts of metal (µmol) in treated (grey bars) and untreated skin (white bars) following exposure to metals in Milli-Q water, 0.5% SLS and ethanol for 2 and 8 h, respectively. Results from single exposure to nickel is shown in the top row. The results for the combined exposure to equimolar amounts of nickel, cobalt and chromium are displayed per metal (middle section) and added (bottom). Data is presented as mean values of from six replicate experiments (*n* = 6, data points) with bars showing the standard deviation. Statistically significant relationships were indicated as (*) for p-values < 0.01 and (**) for p-values < 0.001

Higher amounts of nickel were generally measured in treated skin compared to untreated (Fig. 2, **top**), and the difference was statistically significant for nickel exposure in Milli-Q water. The highest degree of nickel retention was observed for the exposure in ethanol (0.20 and 0.26 µmol for the 2- and 8-hour time-points in treated skin, and 0.16 and 0.22 µmol for the 2- and 8-hour time-points in untreated skin, respectively) followed by exposure in 0.5% SLS and Milli-Q water. The same tendency, however less pronounced, was observed for the 2- and 8-hours skin exposure to nickel in 0.5% SLS (0.07 and 0.12 µmol for the treated skin and 0.06 and 0.08 µmol for the untreated skin) and Milli-Q water (0.09 and 0.11 µmol for the treated and 0.03 and 0.04 µmol for the untreated skin, respectively).

### Co-exposure to nickel, cobalt and chromium in Milli-Q water, 0.5% SLS and ethanol (III)

The proportion of nickel, cobalt and chromium amounts measured in treated as well as untreated skin after 2- and 8 h of exposure respectively, consistently reflected the equimolar conditions of the co-exposure to nickel cobalt and chromium (Fig. 2, **middle**). Similar as to the single exposure to nickel, larger amounts of metal were measured in the treated skin compared to untreated. When the retained amounts of nickel, cobalt and chromium were added, the total amount of metal in skin were found to be at the same level as for nickel single exposure in the cases of exposure in Milli-Q water and ethanol (Fig. 2, **bottom**). For the co-exposure of nickel, cobalt and chromium in 0.5% SLS, the total amounts of metal in skin were instead approximately three times the amounts of that from nickel single exposure and in the same range as for single and co-exposure in ethanol. The difference between metal amounts retained in treated skin was statistically significant only after 8 h of exposure to metals in 0.5% SLS (0.29 and 0.17 µmol Ni + Co + Cr in treated vs. untreated skin) while in ethanol the significance was obtained for both 2- and 8 h of exposure (0.07 and 0.16 µmol Ni + Co + Cr after 2 h and 0.13 and 0.27 after 8 h exposure in treated and untreated skin respectively). For metal co-exposure in Milli-Q water, there were similar amounts of the individual metals measured in skin after 2 h (0.01–0.02 µmol), while the treatment of skin resulted in higher, although not statistically significantly higher, amounts of total metals in skin after 8 h (0.12 µmol Ni + Co + Cr in treated compared to 0.06 µmol Ni + Co + Cr in untreated skin). More information on the individual amounts of Ni, Co, and Cr retained after co-exposure can be found in Supplementary Material Table S2.

### Linear regression with log-transformed retained metal amounts

Among the independent variables tested, SLS pre-treatment, the exposure solvent (Milli-Q water, 0.5% SLS and ethanol), the exposure time and the metal combination have shown to affect the retention of nickel in skin in a statistically significant manner (Supplementary Material Table S3). Moreover, nickel skin retention is affected negatively (coefficient − 0.997) when in the presence of cobalt and chromium, meaning that the presence of other metals in the co-exposure results in lower nickel retention in skin, although the total metal content (Ni + Co + Cr) was higher (see also Fig. 2, bottom row).

In the linear model skin thickness and TEWL (see also Supplementary Material Figure S3 and S4) did not have a statistically significant effect on the nickel skin retention. The model analysis thus indicates that TEWL is not a good predictor of metal in the skin.

## Discussion

The present study demonstrates how the skin’s ability to resist exposure and retain allergenic metals is affected by exposure conditions mimicking intensive hand hygiene practices using water, soap and hand sanitizer and impaired barrier properties. First, we found that experimental treatment of piglet skin with 5% SLS efficiently alters the barrier integrity by means of TEWL. By adopting an established OECD method for skin absorption, we then conducted in vitro experiments that confirmed the SLS treatment consistently facilitated nickel skin penetration, and that exposure to single nickel in ethanol resulted in the highest amount of nickel in skin, compared to that from exposure in Milli-Q water or 0.5% SLS. Finally, co-exposure to nickel, cobalt and chromium in Milli-Q water, 0.5% SLS or ethanol respectively, showed that the amount of metal measured in the skin reflected the equimolar conditions upon exposure and that none of the metals penetrated or retained in the skin more readily than the other. Furthermore, following metal co-exposure in Milli-Q water and ethanol, the metal amount detected in skin added up to similar levels as observed for exposure to nickel only, while for the exposure in 0.5% SLS, the total amount of metal measured in skin doubled.

Impairing the barrier properties of skin using SLS is recommended by OECD TG 439 for in vitro skin irritation [28] and was previously used e.g., in vivo to cause irritation in a study of skin deposition and penetration of nickel [31]. In the present case, SLS treatment of skin was the preferred option since it was considered to additionally contribute to exposure conditions aimed to mimic the effect of hand hygiene practices. SLS concentrations in consumer products typically ranges from 0.01 to 50% in cosmetic products and 1–30% in cleaning products [30]. In a series of experiments, we investigated at which concentration the SLS treatment was most effective with respect to changed barrier properties and thus increased TEWL. We found that 5% SLS was more effective than treatment with 10% SLS, despite a relatively large variability among the 8 replicates from two different piglet individuals (Table 2). The TEWL measure reflects stratum corneum integrity, i.e., the main barrier for permeation resistance, and serves as a predictor of solvent permeation [35]. However, it does not seem to be a good predictor of metal retention in skin, as no correlation was observed between measured amounts of metal in skin and the degree of TEWL changes (Supplementary Material Figure S4). Alternative measures, in human skin, of natural moisturizing factor (NMF) and IL-1α are promising markers for other types of barrier properties such as permeation in deeper skin layers and inflammatory parameters [10], but more research is needed to determine their usefulness as a predictor for skin uptake of allergenic metals.

The results from exposure of untreated and treated skin, confirmed that the SLS pre-treatment enhances penetration of nickel and higher amounts of nickel were retained in skin compared to the untreated case. This is in line with previous findings on irritancy and skin damage caused by SLS as evaluated by several methods including the TEWL measure [9, 36, 37] and the ability of SLS to enhance permeation of other compounds [38–40]. Although the exact mechanism of SLS on skin barrier function has not been clarified, studies have pointed to delipidization [41, 42], morphological changes of corneocytes [43], or to damage to the deeper nucleated layers of the epidermis [44, 45]. These changes to the lipid lamellae organization may have contributed to the higher increase of nickel into the SLS treated skin.

Also, water causes skin irritancy and disruption similar to that of surfactants [11] but no study to our knowledge have investigated the permeation enhancing capacity of water. Since the results of nickel in untreated skin from exposure in Milli-Q water is the lowest that we observe in our experiments, it is anticipated that the SLS, both the pre-treatment and the exposure to 0.5% SLS have a larger influence on metal penetration and retention in skin than the other tested exposure solvents. The highest measured levels in the skin were observed for exposure to nickel in ethanol, which at the same time showed a large variation between the repeated experiments. This can be partially explained by inter-individual skin differences rather than the ethanol itself, indicated also by the TEWL values observed (Table 2, Supplementary Material Figure S4). In addition, ethanol interacts with stratum corneum lipids [46] and is known to be a skin permeation enhancer [47], which could contribute to the explanation of the high nickel levels measured in the skin after ethanol exposure.

When comparing our results with human nickel penetration studies, a major limitation is that human volunteer studies only investigate nickel penetration in stratum corneum, and varying number of layers of stratum corneum as the commonly used method tape-stripping is difficult to standardise. However, human volunteer data do indicate that nickel penetrates beyond the stratum corneum more efficiently in cases of either a filaggrin mutation or pre-treatment of skin with SLS [31, 48]. The fact that we find more nickel in skin after SLS treatment than in the reported human studies, is likely a result of analysing the entire skin, and not only the stratum corneum. When comparing our data to other in vitro studies, using human skin, nickel powder has been shown to penetrate damaged skin to a higher extent, together with also cobalt and chromium powders [49]. Furthermore, nickel has been shown to quickly penetrate beyond the stratum corneum in skin after exposure using various methodologies. The results from an in vitro study using nickel salts [50] have shown that less nickel is recovered from the stratum corneum surface as the exposure time increases. Furthermore, findings from tape-stripping [51] and imaging mass spectrometry [52], indicate that nickel can penetrate the deepest layers of the stratum corneum and enter the upper living epidermis.

The measured amounts of nickel, cobalt and chromium in the skin after combined exposure showed proportionality with the equimolar composition of the metals upon exposure and thus no possible preferential retention of the allergenic metals could be demonstrated. Results show that metal penetration occur in a time-dependent manner, which is in line with previous observations of simultaneous exposure to several metals [23]. The same study reported that the sum of metals in co-exposure (Ni, Co and Cr) resulted in higher metal amounts measured in skin compared to their single-metal-exposure counterparts, a tendency that was observed only for the metal co-exposure in 0.5% SLS in the current study. For exposures in Milli-Q water and ethanol, i.e., without surfactant present, the sum of metals from co-exposure is similar to the amount of nickel in the single metal exposure case. This finding indicates the possibility that the skin’s ability to retain metals has a saturation limit determined by the status of the skin barrier and the magnitude of the dose, in other words, infinite or finite conditions.

The present study focussed on the skin retention of nickel under different exposure conditions and skin status. A disadvantage of this study design is that, for various reasons including time and resources, metal concentrations in the receptor have not been quantified. With the information on percutaneously absorbed amounts, a better understanding of the skin’s barrier properties and ability to retain the metals that penetrated stratum corneum, could have been obtained. Having studied the single exposures to cobalt and chromium would as well have contributed to understanding potential co-exposure effects also for these metals. Another obvious limitation of the current study is the number of replicated experiments (*n* = 6) and the number and distribution of piglet individuals exposed to metals (*n* = 18), where a larger scale would of course be desirable in order to control for inter-individual variations.

This study is occupationally relevant as it demonstrates that a damaged skin barrier can absorb metals into the stratum corneum and deeper layers. This finding is particularly important for the many occupational groups working with both metals and wet work. It is known that nickel, cobalt and to some extent also chromium allergy is prevalent in, for example, car mechanics, electroplaters, health care workers, cement workers, hard-metal workers and electronic workers [53–59]. Common exposure factors in these occupations are direct contact with metals in skin on hands, coupled to extensive cleaning of hands with water, soap and sometimes even abrasive creams. Therefore, careful attention to prevention is warranted in these types of occupations with regards to metal exposure and hand hygiene practices. Recommendations of hand hygiene practices could be developed into specific guidelines taking into consideration each case where the skin needs protection. This has to be done on a case-by-case approach since the exposure scenarios may vary substantially.

## Conclusion

In this study, we have demonstrated that an SLS treatment of skin alters the skin barrier properties with regards to TEWL. Furthermore, we have investigated differences in nickel retention between treated and untreated skin and how it is affected by exposure to other allergenic metals and continued skin-altering treatment mimicking intensive hand hygiene practices in the form of water, a surfactant and ethanol. In all investigated exposure cases, the treated skin is subject to higher level of metal retention. The exposure to nickel in ethanol and combined exposure to metals in 0.5% SLS, respectively, constitute the most severe scenarios, leading to the highest metal retention cases. These findings are important, as they show that hygiene practices could lead to an increased retention of metals in the skin. This highlights the need for suitable skin protection practices that do not disrupt the skin barrier, especially for those occupations with a high metal exposure or wet work exposure in combination with metals exposure. Furthermore, future research should be focused on elaborating these findings in occupationally exposed worker cohorts, to validate the results of the study in real-life settings.

## Electronic supplementary material

Below is the link to the electronic supplementary material.

Supplementary Material 1

## Data Availability

No datasets were generated or analysed during the current study.
