# Supplementary material for: Retention of nickel, cobalt and chromium in skin at conditions mimicking intense hand hygiene practices using water, soap, and hand-disinfectant in vitro
Source: J Occup Med Toxicol. 2024 Nov 6;19:44. doi: 10.1186/s12995-024-00442-5 (PMC11539800; doi:10.1186/s12995-024-00442-5)

Retention of Nickel, Cobalt and Chromium in skin at conditions mimicking intense hand hygiene practices using water, soap, and hand-disinfectant *in vitro*

Authors: Libe Vilela^1^, Linda Schenk^1^, Anneli Julander^1,2^, Klara Midander^1,2^

1. Institute of Environmental Medicine (IMM), Karolinska Institutet, Stockholm, Sweden

2. IVL Swedish Environmental Research Institute, Stockholm, Sweden

## Corresponding author:

Libe Vilela, Karolinska Institutet, Stockholm, Sweden; and email: [libe.vilela.2@ki.se](mailto:libe.vilela.2@ki.se), phone: +46-(0)76 447 30 87


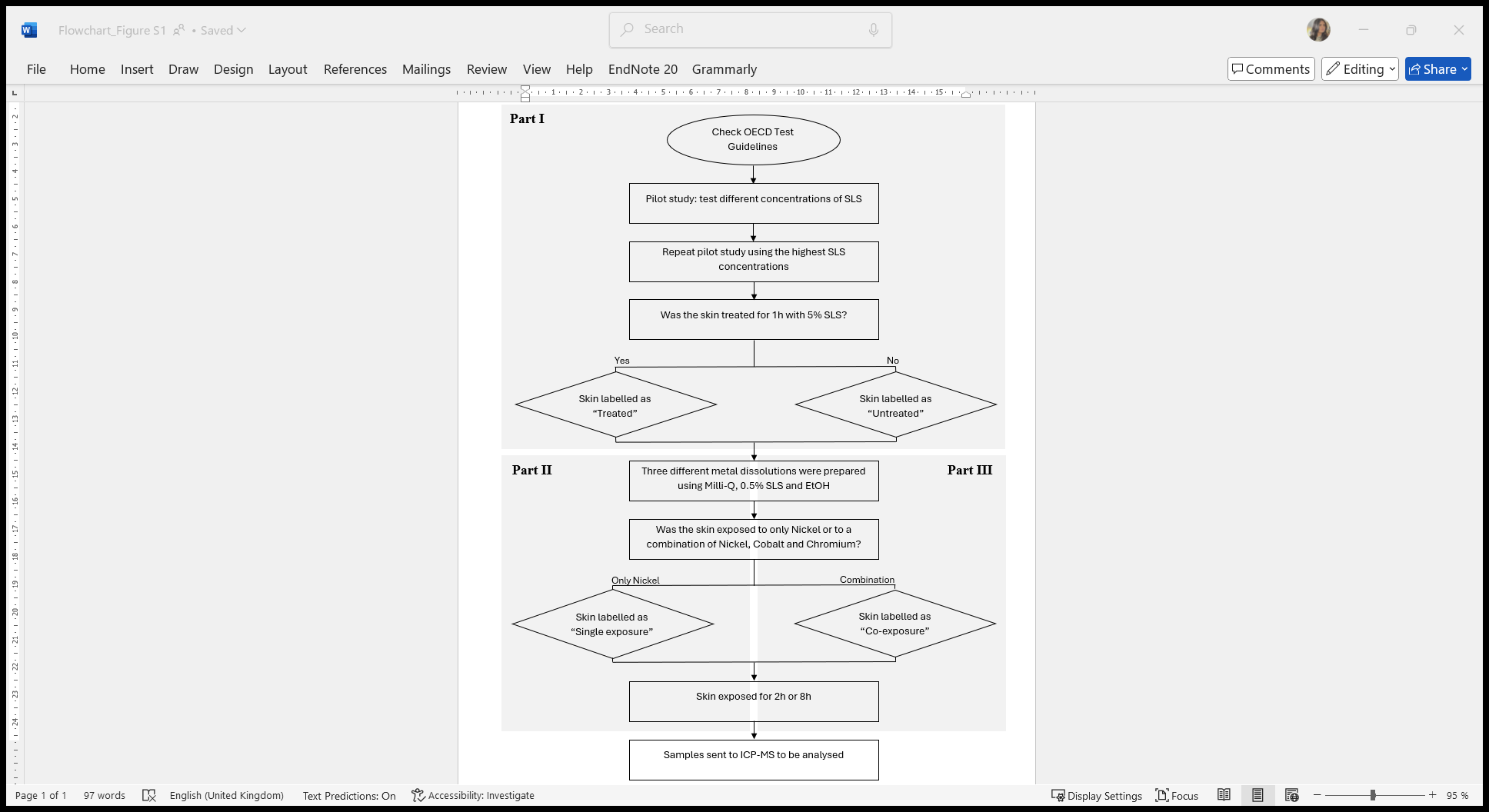


**Figure S1**. Flowchart describing how the different parts of the study evolved with time.

**Table S1.** Median TEWL values (g×m^-2^×h^-1^) recorded to assess the barrier integrity of piglet skin after each step of the *in situ* procedure aiming to simulate experimental exposure conditions affected by intense hand hygiene practices (four repeated measurements, 20 minutes after each step). The range of these repeated measurements, somewhat indicative of intra- and inter individual variations, is represented by min and max values. a) Stillborn piglets (n=4) were *in situ* rinsed with lukewarm water at arrival to the laboratory to e.g., remove any dirt. b) Warm water and soap were applied to the piglet’s back and flank following hand hygiene practice behavior, i.e. apply soap on the skin and rinse it off with warm water and repeat for 5 minutes. c) A total of 25 pumps of hand disinfectant were applied on the piglet’s back and flank, leaving the product to “air-dry” before the next dose/pump was applied on the skin. d) Washed skin was excised and frozen for storage at -20 ºC. After the steps a), b), and c) the piglet skin was pat dried using paper wipes to ensure that no residual water on the skin altered the TEWL measurements.

| **Steps**  **(TEWL measured 20 min after each step)** | | **Median TEWL**  **(Min; Max)** |
| --- | --- | --- |
| *Preparation of skin for experiment* | | |
| a) | After rinsing with lukewarm water *in situ* | 7.65  (5.8; 10.6) |
| *Simulating intense hand hygiene practices* | | |
| b) | After warm water and soap, 5 min, *in situ* | 6.15  (4.9; 8.5) |
| c) | After hand disinfectant application, 25 times, *in situ* | 5.95  (3.5; 11.4) |
| d) | Excised skin | 5.25  (3.8; 7.1) |
| *Skin stored in freezer* | | |

| **Single exposure** | **Ni (µg)** | 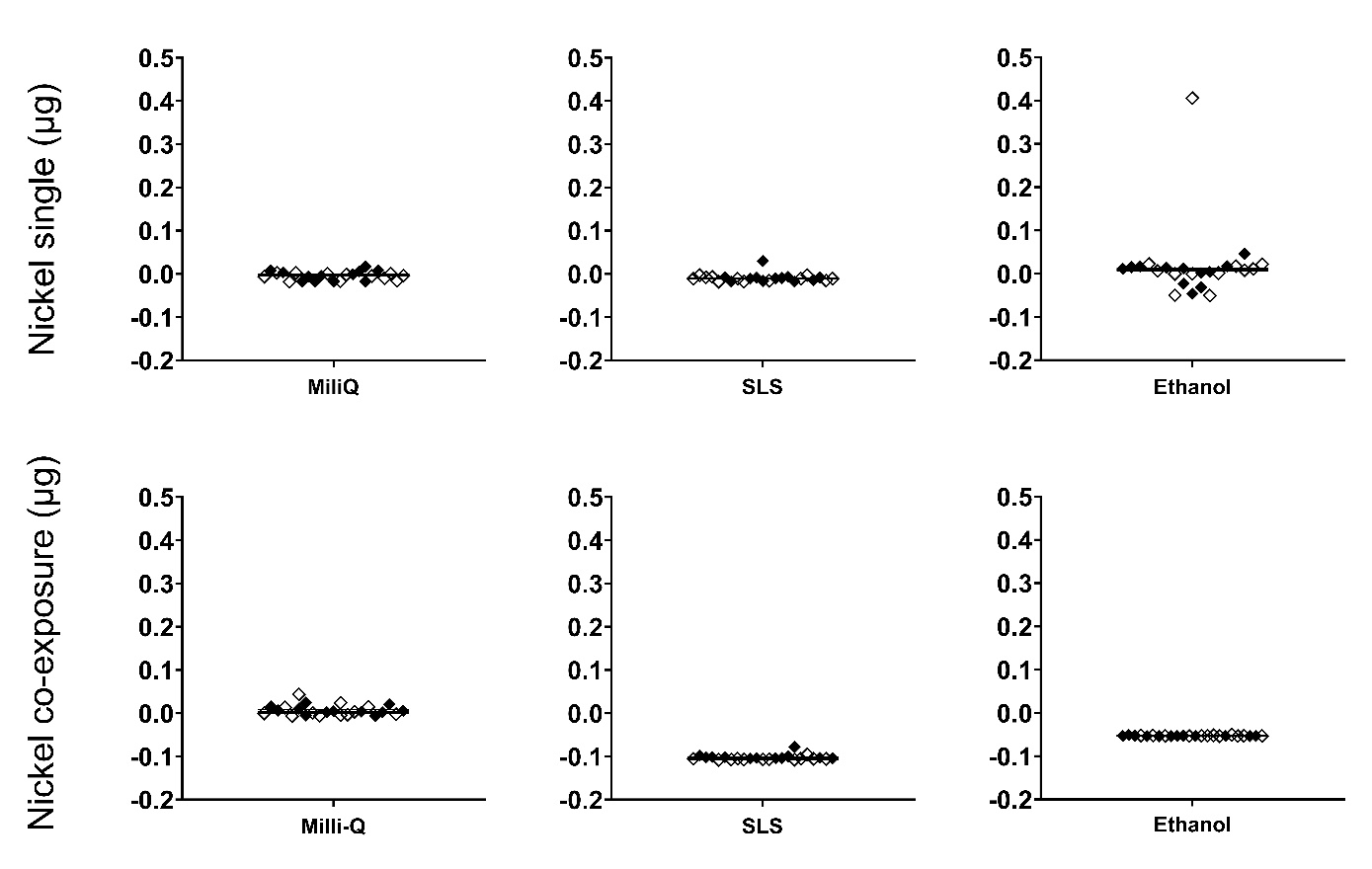 |
| --- | --- | --- |
| **NiCoCr co-exposure** | **Ni (µg)** | 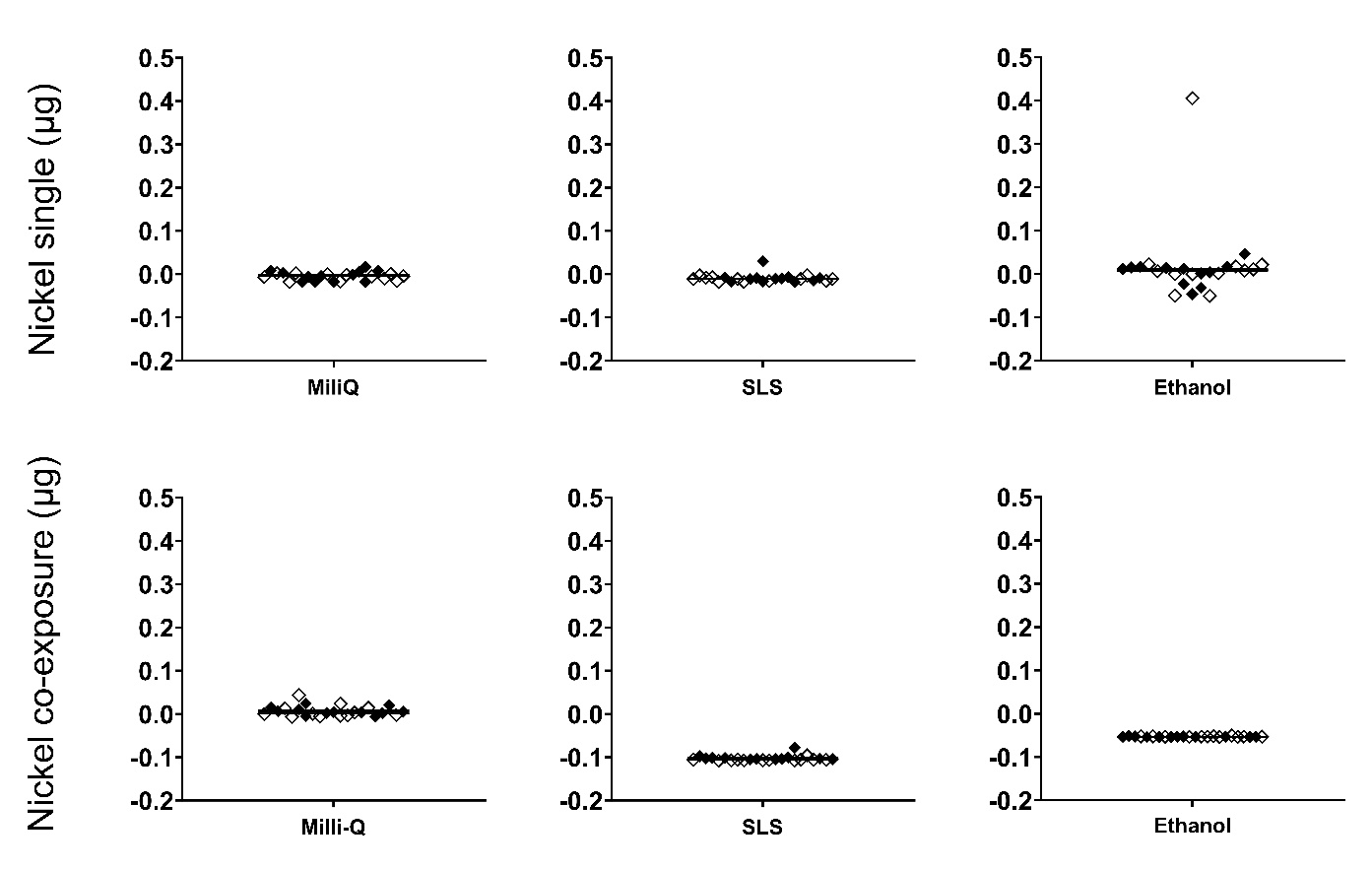 |
|  | **Co (µg)** | 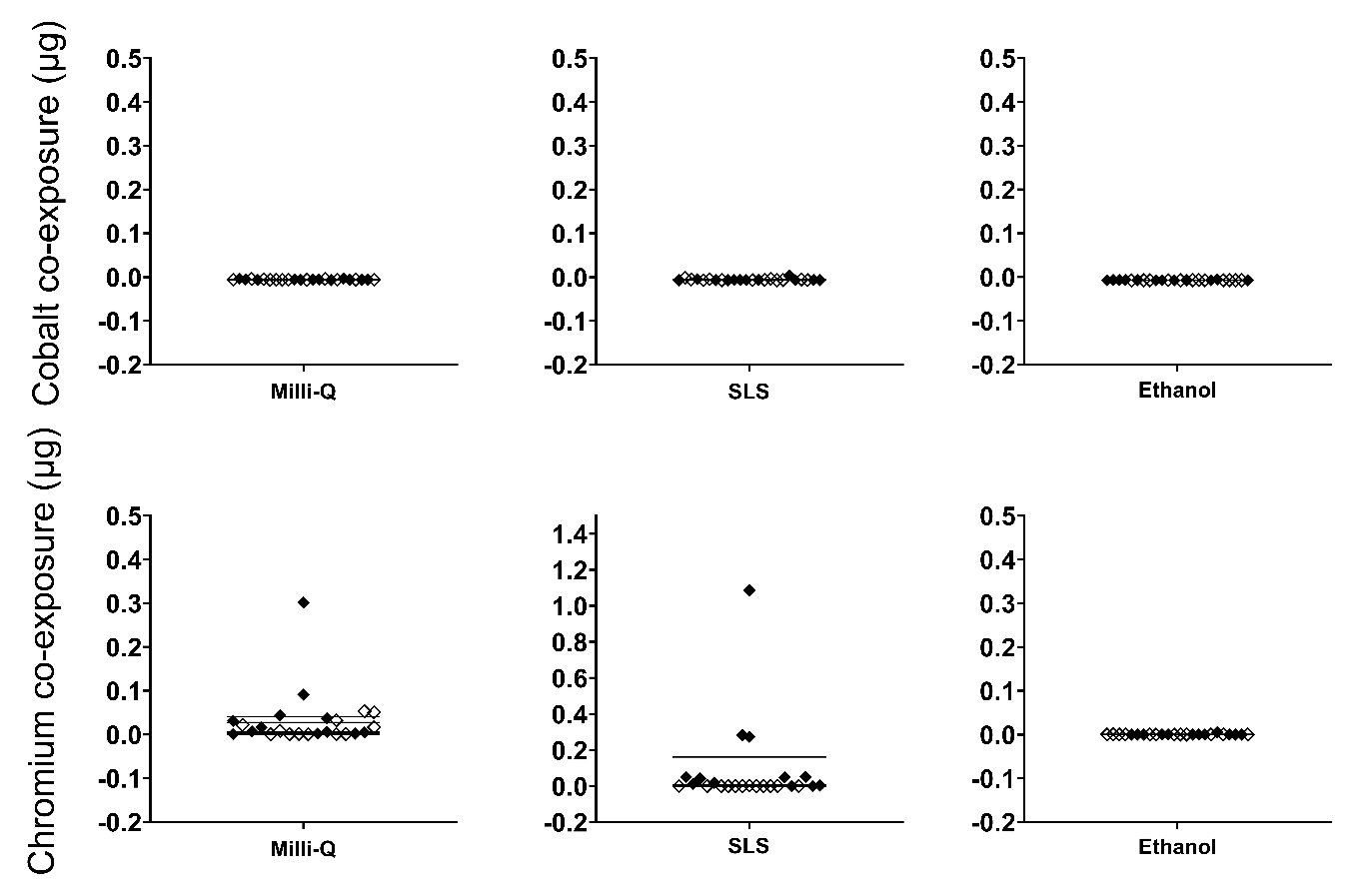 |
|  | **Cr (µg)** | 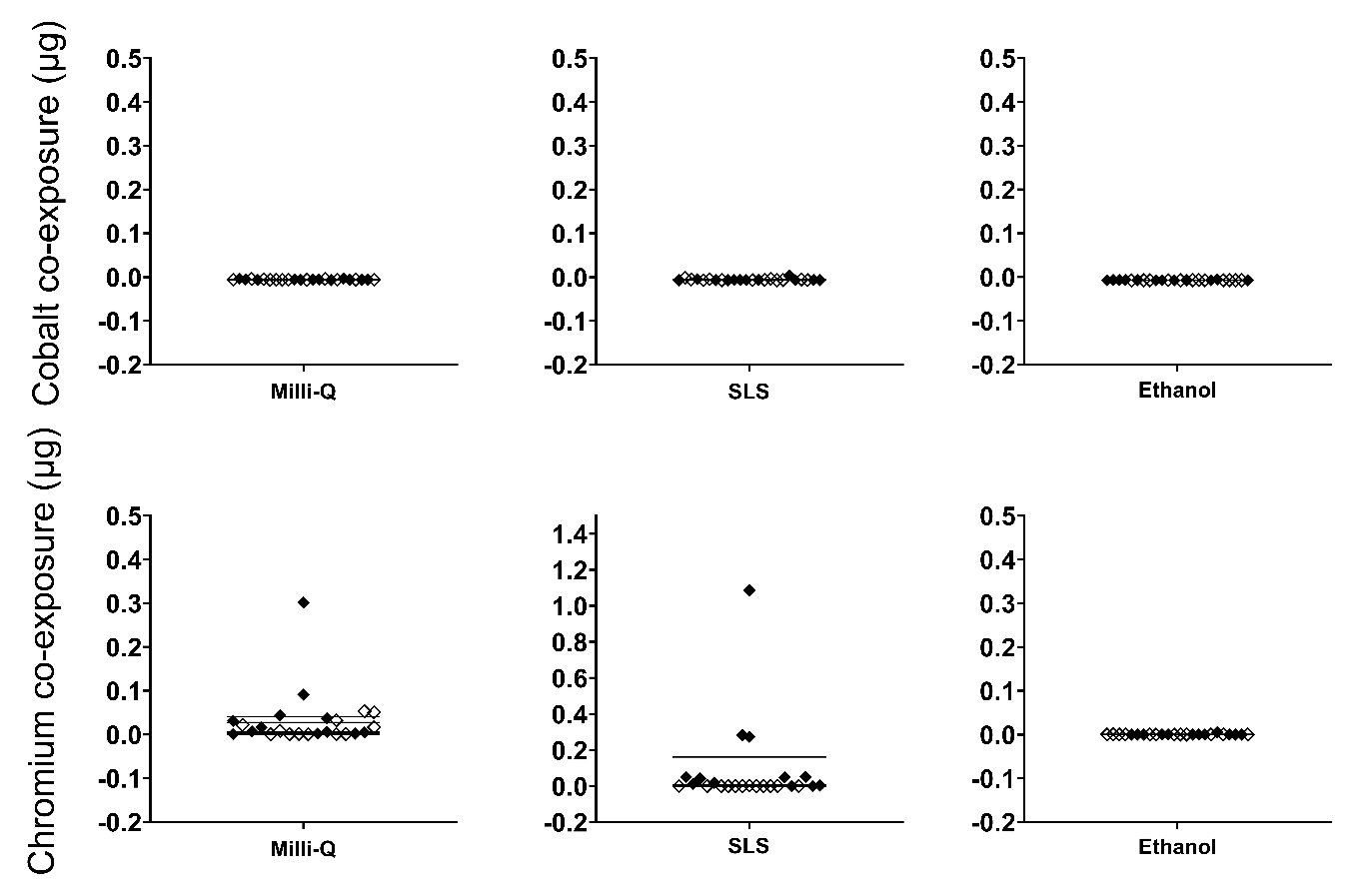 |

**Figure S2**. Results from blank exposures, i.e., negative control samples, performed to evaluate any metal background in the experiments. Skin uptake profiles (µg) during single exposure to nickel and co-exposure of nickel, cobalt, and chromium. Both treated and untreated skin pieces are plotted together. Blank exposures were created for each exposure scenario of metal and the exposure solvents Milli-Q water, 0.5% SLS and ethanol respectively. Line corresponds to median value, white rectangle is 2 hours exposure and black rectangle is 8 hours exposure and there are in total six replicates (n=6, data points).

**Table S2.** Measured amounts of individual Ni, Co and Cr (µmol) in treated and untreated skin following co-exposure to Ni+Co+Cr in Milli-Q water, 0.5% SLS and ethanol for 2 and 8 hours respectively.

|  |  | **2h - Untreated** | **2h - Treated** | **8h - Untreated** | **8h - Treated** |
| --- | --- | --- | --- | --- | --- |
| **Nickel** | **Milli-Q** | 0.016 | 0.014 | 0.020 | 0.038 |
|  | **0.5% SLS** | 0.037 | 0.048 | 0.051 | 0.095 |
|  | **Ethanol** | 0.025 | 0.054 | 0.037 | 0.090 |
| **Cobalt** | **Milli-Q** | 0.017 | 0.014 | 0.020 | 0.040 |
|  | **0.5% SLS** | 0.039 | 0.051 | 0.062 | 0.101 |
|  | **Ethanol** | 0.026 | 0.056 | 0.041 | 0.096 |
| **Chromium** | **Milli-Q** | 0.014 | 0.011 | 0.019 | 0.047 |
|  | **0.5% SLS** | 0.033 | 0.048 | 0.057 | 0.096 |
|  | **Ethanol** | 0.024 | 0.048 | 0.048 | 0.083 |

**Table S3.** Linear regression analysis of log-transformed nickel skin retention (dependent variable). Characteristics of the model (coefficient, 95% Confidence interval (CI), and p-value) are shown in the table below (R^2^ value = 60.23% and adjusted R^2^ value = 58.18%). Residual standard error = 0.63021 on 136 degrees of freedom).

|  | Coefficient | 95% CI | p-value |
| --- | --- | --- | --- |
| Thickness | -0.63 | -1.43 - 0.19 | 0.13 |
| SLS treatment:  · Untreated  · Treated | -------  0.49 | --------  0.22 - 0.76 | --------  0.000557 *** |
| Exposure solvent:  · Milli-Q water  · 0.5% SLS  · EtOH | --------  0.76  0.97 | --------  0.51 - 1.01  0.73 - 1.22 | --------  1.15e-8 ***  7.60e-13 *** |
| Exposure time:  · 2h  · 8h | --------  0.48 | --------  0.28 - 0.68 | --------  4.74e-06 *** |
| Metal combination:  · Ni single  · Ni+Co+Cr | --------  -0.997 | --------  -1.21 - -0.78 | --------  1.06e-15 *** |
| TEWL | 0.004 | -0.03 - 0.04 | 0.812 |

(***) indicates p-values lower than 0.001.

**Figure S3.** Measured amount of metal in skin versus measured skin thickness (n=144). Results show that skin thickness does not affect the uptake of nickel into the skin. White markers indicate untreated piglet skin while black markers indicate SLS-treated skin. Nickel in single exposure (square shape) and nickel in co-exposure with cobalt and chromium (triangle shape). The results for the 8 samples with metal content above 15 nickel µg came from the same two piglet individuals.


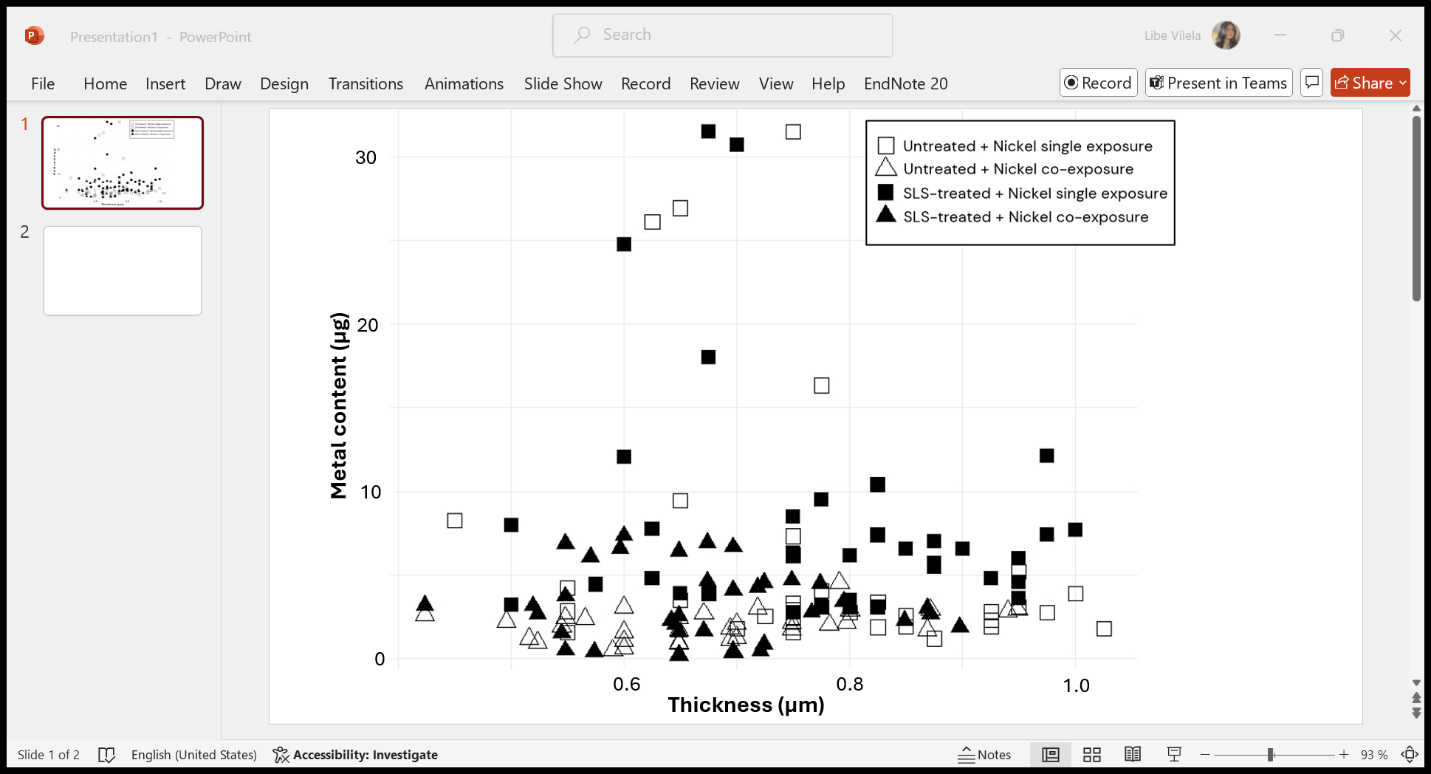


**Figure S4.** Measured amount of metal in skin versus the average of last measured TEWL values of skin (n=144). Results show that TEWL does not seem to be an appropriate parameter to predict skin retention of metals in skin. White markers indicate untreated piglet skin while black markers indicate SLS-treated skin. Nickel in single exposure (square shape) and nickel in co-exposure with cobalt and chromium (triangle shape). The results for the 8 samples with metal content above 15 nickel µg came from the same two piglet individuals.


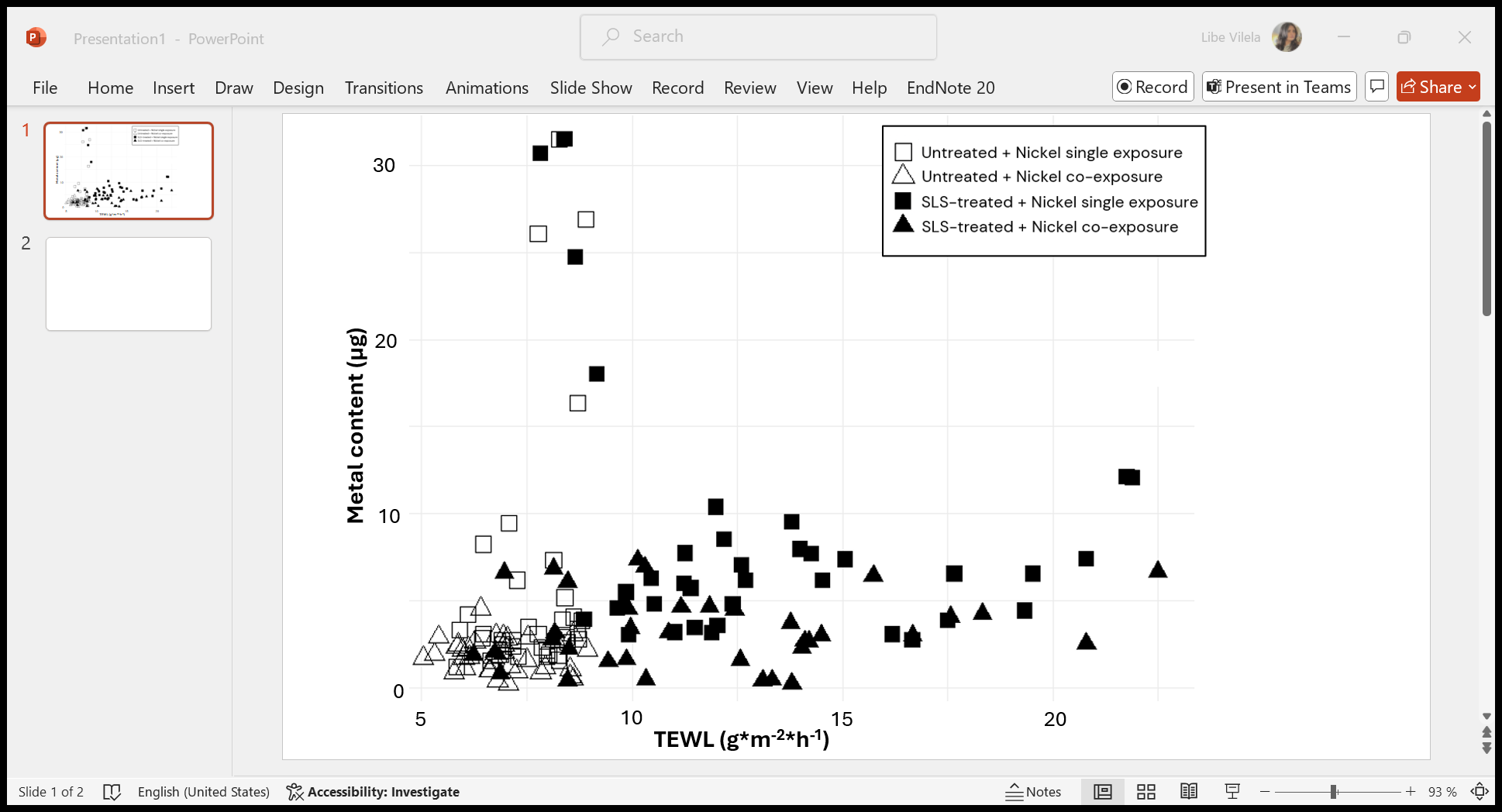

Supplement: Supplementary file 1 — Supplementary Material 1 [file 12995_2024_442_MOESM1_ESM.docx]
